# Blockwise PPG Enhancement Based on Time-Variant Zero-Phase Harmonic Notch Filtering

**DOI:** 10.3390/s17040860

**Published:** 2017-04-14

**Authors:** Chanki Park, Hyunsoon Shin, Boreom Lee

**Affiliations:** 1 School of Mechanical Engineering, Gwangju Institute of Science and Technology (GIST), Gwangju 61005, Korea; chanki@gist.ac.kr; 2 Emotion Recognition IoT Research Section, Hyper-connected Communication Research Laboratory, Electronic and Telecommunications Research Institute (ETRI), Daejeon 34129, Korea; hsshin@etri.re.kr; 3 Department of Biomedical Science and Engineering (BMSE), Institute of Integrated Technology (IIT), Gwangju Institute of Science and Technology (GIST), Gwangju 61005, Korea

**Keywords:** photoplethysmogram, motion artifact, single-input, forward-backward filtering, linear time-variant

## Abstract

So far, many approaches have been developed for motion artifact (MA) reduction from photoplethysmogram (PPG). Specifically, single-input MA reduction methods are useful to apply wearable and mobile healthcare systems because of their low hardware costs and simplicity. However, most of them are insufficiently developed to be used in real-world situations, and they suffer from a phase distortion problem. In this study, we propose a novel single-input MA reduction algorithm based on time-variant forward-backward harmonic notch filtering. To verify the proposed method, we collected real PPG data corrupted by MA and compared it with existing single-input MA reduction methods. In conclusion, the proposed zero-phase line enhancer (ZLE) was found to be superior for MA reduction and exhibited zero phase response.

## 1. Introduction

As the population has aged, healthcare expenditures have accelerated explosively over the last decade [1]. Alongside the expansion of the healthcare industry and advances in information and communication technology (ICT), ubiquitous healthcare (u-healthcare) has been a particular concern. Although many u-healthcare techniques have been developed, fast and accurate data processing as well as mobility still remain as challenges [2]. Recently developed ICT devices, such as smart phones [3], smart watches [4], and smart mirrors [5], employ a photoplethysmogram (PPG) to acquire the user’s vital signs.

**Figure 1:**
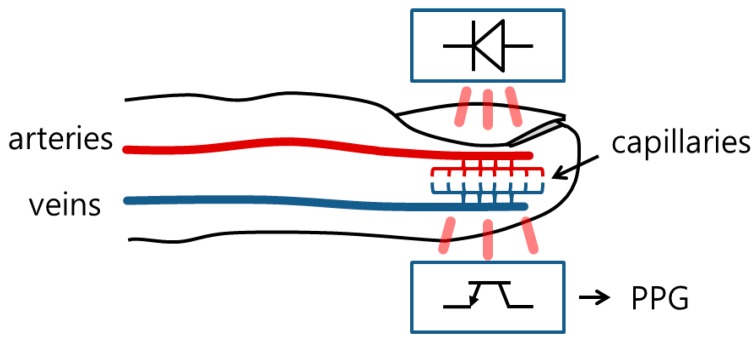
Principle of PPG measurement.

The PPG can be measured from the intensity of transmitted infrared (or red) light through a finger, as shown in Figure 1. Because the PPG indicates the absorbance of blood and tissue, it provides a variety of health information such as heart rate (HR), respiratory rate (RR) [6], heart rate variability (HRV) [7], SpO2, and arterial blood pressure [8,9]. Furthermore, it is conveniently measured using only a single-channel non-invasive sensor.

In spite of the PPG’s usefulness, it is difficult to use in mobile applications because it is easily contaminated by motion artifacts (MA) and the feasible frequency ranges of PPG and MA overlap each other. Hence, many MA reduction algorithms have been developed. MA reduction techniques can be roughly categorized as either single-input MA reduction methods or multiple-input MA reduction methods.

The conventional multiple-input MA reduction method is an adaptive noise canceller (ANC) [10]. The ANC requires two input signals: one is contaminated PPG, and the other is the reference signal acquired from the noise field. The ANC not only exhibits good performance, but also is an on-line processor. It is assumed in idealizations that the real MA and the reference signal are perfectly correlated. However, these two signals are generally uncorrelated in real situations, leading to disturbance [11]. Another conventional approach is independent component analysis (ICA) [12,13]. This method separates mixed multiple signals into two independent components: a clean PPG and MA. It offers satisfactory performance, but the user must manually select the clean PPG from independent components; additionally, its output scale is ambiguous. Recently, to solve these problems, a hybrid version of the ICA that combines a constrained ICA with an adaptive filter was proposed [14]. It automatically selects the clean PPG by means of a synthetic reference signal and compensates for the scale ambiguity using an adaptive filter.

Single-input MA reduction methods offer low hardware costs and complexity [15]. Hence, single-input MA reduction methods are more suitable for u-healthcare devices than multiple-input MA reduction methods. One of the conventional single-input MA reduction methods is the wavelet de-noising method. It decomposes the input signal into several level coefficients and eliminates the coefficients that correspond to noise criterions [16]. The wavelet is not only a good de-noising tool but also serves as a time-frequency analyzer. Cycle-by-cycle Fourier series analysis (CFSA) was proposed by K. A. Reddy et al. [17]. The CFSA segments input signals at each cycle and applies a Fourier series to each segmented pulse. By means of the integrals of the Fourier series, MA components are cancelled out; as a result, the CFSA offers outstanding performance with respect to the accuracy of peaks of filtered PPG [18]. However, CFSA requires accurate knowledge of the period of each cycle. Recently, the singular spectrum analysis (SSA) based MA reduction approach was proposed [19]. It features better performance than time-domain ICA without any additional input signal, but its computational burden is considerable because SSA requires an eigendecomposition procedure. Several approaches based on digital filtering had been developed to eliminate interferences in bio-signals [20,21,22] requiring only single input signal. Specifically, Lee et al. proposed the adaptive comb filter (ACF), which enhances a quasi-periodic component using a time-variant harmonic infinite impulse response (IIR) notch filter [22]. Since the dominant frequency of MA is generally adjacent to that of PPG, the frequency response of notch filter needs to be adequately sharp to distinguish PPG from MA. ACF occasionally showed quite remarkable performance when it found the fundamental frequency of PPG accurately and filtered the signal with sharp frequency response. On the other hand, if the estimated frequency is not matched with the target frequency, then ACF brings a phase distortion problem. Even though it had very high potential with respect to eliminating MA, ACF did not show promising performance generally because there are limits to accurate frequency estimation and consecutive phase distortion problem. In this paper, we propose a novel MA reduction algorithm based on time-variant forward-backward harmonic IIR notch filtering that solves the phase distortion problem of the ACF and call it Zero-phase Line Enhancer (ZLE). The ACF and ZLE are explained in detail in the next section, and specifically we proof the zero phase response property of ZLE. In the results section, we compare the ZLE with existing single-input MA reduction methods using real-world PPG data. In the discussion section, the experimental results are interpreted and discussed.

## 2. Methods

### 2.1. Adaptive Comb Filter

**Figure 2:**
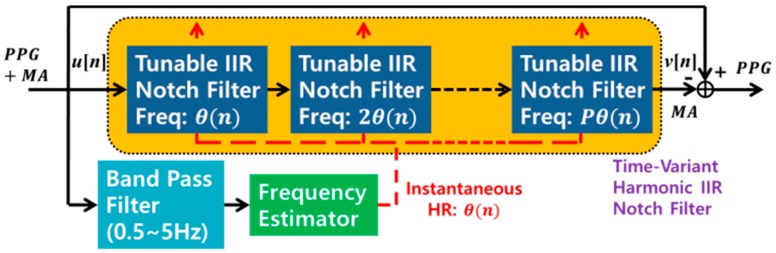
ACF with harmonic IIR notch filter.

In this subsection, we briefly review the ACF, which enhances a PPG buried in MA. The ACF is suitable for wearable and mobile devices because it is an on-line processor and it requires only a single-input measurement. As can be observed in Figure 2, ACF involves two steps: (i) instantaneous HR estimation using a frequency estimator and (ii) by means of a harmonic IIR notch filter [6], enhancement of the quasi-periodic component (clean PPG) with the instantaneous HR estimated in the first step.

In this study, an adaptive lattice notch filter (ALNF) was used as the frequency estimator in the first step of the ACF. ALNF is an adaptive IIR notch filter combined with a lattice structure. Because ALNF has less computational complexity as well as high stability [23], it is frequently used in many signal processing applications. All procedures are explained in [24] in detail.

In the second step of the ACF, the quasi-periodic component, of which the HR is the fundamental frequency, can be separated by the harmonic IIR notch filter as follows [25]:

(1) $$H\left(z,\theta \right)=\overset{P}{\underset{j=1}{\prod }}\frac{1+r_{j}}{2}\frac{1-2\cos\left(j\theta \right)z^{-1}+z^{-2}}{1-\left(1+r_{j}\right)\cos\left(j\theta \right)z^{-1}+r_{j}z^{-2}}\;=\overset{P}{\underset{j=1}{\prod }}k_{j}\frac{1+b_{1j}z^{-1}+z^{-2}}{1-a_{1j}z^{-1}-a_{2j}z^{-2}}$$

where *a*_1*j*_ = (1 + *r_j_*)cos(*jθ*), *a*_2*j*_ = −*r_j_*, *b*_1*j*_ = −2cos(*jθ*), and *k_j_* = (1 + *r_j_*)/2. *j* represents the order of harmonics. *jθ* represents the *j*-th notch frequency, and *θ* is the fundamental frequency (instantaneous HR). The parameter *P* is the number of harmonic components, and *r_j_* is the pole-zero contraction factor (0 < *r_j_* < 1). According to a decrease of the value of *r_j_* from 1 to 0, the frequency response of the *j*-th notch filter becomes wider. Because the variance of a high-order harmonic frequency is larger than that of a low-order harmonic frequency, it is necessary to filter out high-order harmonic components using a wide bandwidth filter to address the HR estimation error caused by the variation of HR. Hence, with increasing harmonic order *j*, *r_j_* need to be smaller, so in this study we define *r_j_*, with a positive small value *δ* (typically 0.02~0.04) as follows:

(2) $$r_{j}=1-j\delta$$

The true instantaneous HR is time-varying, so that instead of constant *θ*, the ACF uses time-varying *θ*(*n*) estimated in the first step. The output of the time-variant harmonic IIR notch filter is as follows [26]:

(3) $$v\left[n\right]=\overset{n}{\underset{m=-\infty }{\sum }}h\left[n-m,\theta \left(n\right)\right]u\left[m\right]\;\;=\overset{\infty }{\underset{m=0}{\sum }}h\left[m,\theta \left(n\right)]u[n-m\right]$$

where *v*[*n*], *u*[*n*], and *h*[*m*, *θ*(*n*)] represent the output, the input, and the time-variant impulse response, respectively, of the time-variant harmonic IIR notch filter with parameter *θ*(*n*). Equation (3) can be expressed as a z-transform [27]:

(4) V(z)=H(z,θ(n))U(z)

Because the time-variant harmonic IIR notch filter removes the clean PPG component in the contaminated PPG *u*[*n*], the output *v*[*n*] corresponds to the MA component. Eventually, the clean PPG *y_ACF_*[*n*] is acquired as follows:

(5) $$y_{ACF}\left[n\right]=u\left[n\right]-v\left[n\right]$$

**Figure 3:**
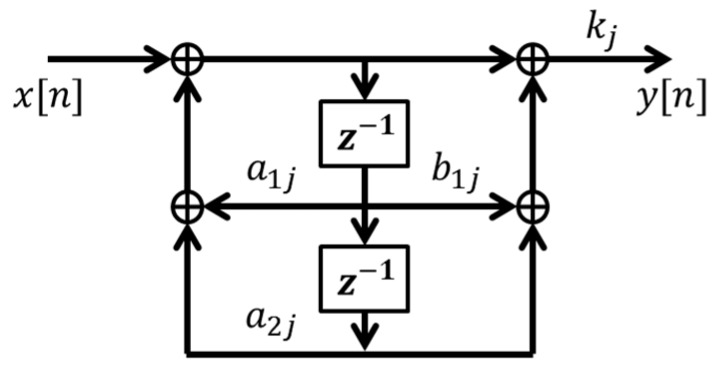
Direct form 2 implementation of *j*-th IIR notch filter of harmonic IIR notch filter. *a*_1*j*_ = (1 + *r_j_*)cos(*jθ*(*n*)), *a*_2*j*_ = −*r_j_*, *b*_1*j*_ = −2cos(*jθ*(*n*)), and *k_j_* = (1 + *r_j_*)/2.

We implemented the time-variant harmonic IIR notch filter by direct form 2 [23] (see Figure 3) and set the number of harmonic components *P* to 8.

### 2.2. Zero-Phase Line Enhancer

**Figure 4:**
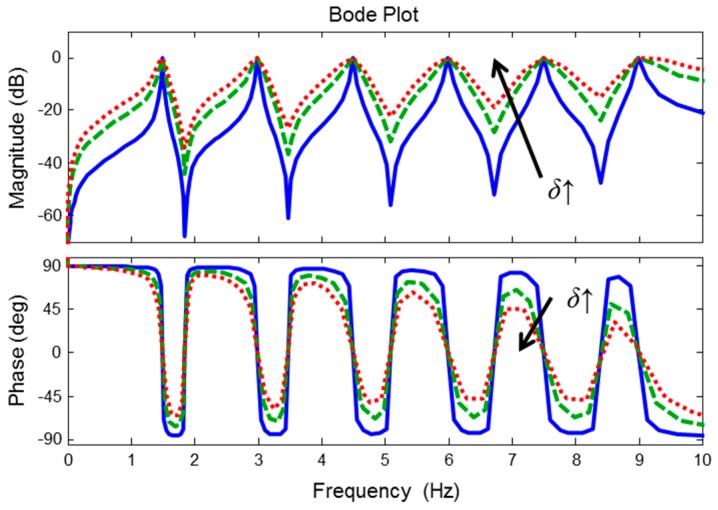
Bode plot for ACF. The shape of the frequency response of ACF is determined by *δ* in Equation (2). The annotation *δ*↑ indicates the increment of the value of *δ.*

**Figure 5:**
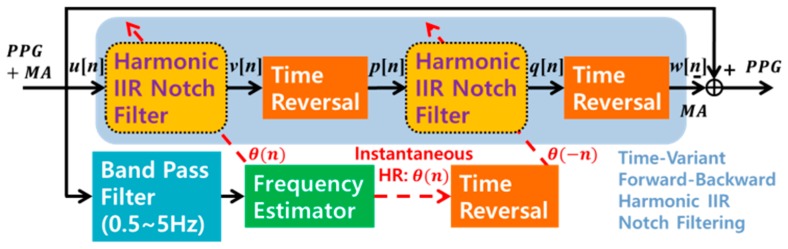
Block diagram representation of Zero-phase Line Enhancer (ZLE).

If the instantaneous HR is exactly estimated, then the ACF is superior at extracting the clean PPG. However, the estimated instantaneous HR is not always the same as the real instantaneous HR, and the estimation error yields a novel phase distortion (see Figure 4). To solve the phase distortion problem of the ACF, we propose the ZLE based on time-variant forward-backward harmonic IIR notch filtering, which has zero phase response (see Figure 5).

A linear phase filter is frequently used in many signal processing fields because it does not yield phase distortion and is easily implemented by a finite impulse response (FIR) filter [28]. However, to satisfy a desired filter response, the FIR filter demands a large number of filter orders and much more computational complexity than the IIR filter. Hence, we employed a forward-backward IIR filter to design ZLE [29]. It filters an input signal both forward and backward in time, so it should store the entire forward filter output *v*[*n*] as well as the input signal *u*[*n*]. As shown in Figure 5, the time-variant forward-backward harmonic IIR notch filtering of ZLE is composed of four sequential steps: (i) time-variant forward filtering, (ii) time reversing, (iii) time-variant backward filtering, and (iv) time reversing. The first step is time-variant harmonic IIR notch filtering, which is identical to the ACF case (see (3)). The second step is the time reversal, which takes the output of the harmonic IIR notch filter *v*[*n*] as the input. The time reversal operator reverses the order of the time sequence of the input signal. For convenience, we denote the output of the time reversal operator *v*[−*n*] by *p*[*n*]. We can write the z-transform of *p*[*n*] as

(6) $$P\left(z\right)=V^{*}\left(1/z^{*}\right)\;=H^{*}\left(1/z^{*},\theta \left(-n\right)\right)U^{*}\left(1/z^{*}\right)$$

The superscript * represents the complex conjugate. In the third step, the time-variant harmonic IIR notch filter is employed again with input *p*[*n*], and its parameter was assigned to *θ*(−*n*) because its time-variant impulse response should be time reversed. This process means time-variant backward filtering. The output of this step is as follows:

(7) $$q\left[n\right]=\overset{n}{\underset{m=-\infty }{\sum }}h\left[n-m,\theta \left(-n\right)\right]p\left[m\right]\;\;=\overset{\infty }{\underset{m=0}{\sum }}h\left[m,\theta \left(-n\right)]p[n-m\right]$$

where *q*[*n*] is the output of the third step and its z-transform *Q*(*z*) and frequency response *Q*(*e^jω^*) are written as

(8) $$Q\left(z\right)=H\left(z,\theta \left(-n\right)\right)P\left(z\right)\;=H\left(z,\theta \left(-n\right)\right)H^{*}\left(1/z^{*},\theta \left(-n\right)\right)U^{*}\left(1/z^{*}\right)$$

(9) $$Q\left(e^{j\omega }\right)={\left|H\left(e^{j\omega },\theta \left(-n\right)\right)\right|}^{2}U^{*}\left(e^{j\omega }\right)$$

In the last step, the time reversal operator is used again to reverse the order of the time sequence of *q*[*n*]. Thus, the output of the last step *w*[*n*] is *q*[-*n*], and its frequency response is

(10) $$W\left(e^{j\omega }\right)={\left|H\left(e^{j\omega },\theta \left(n\right)\right)\right|}^{2}U\left(e^{j\omega }\right)$$

As shown in (10), the time-variant forward-backward harmonic IIR notch filtering has zero phase response, and its output correspond to the MA component similar to the ACF. Finally, we can obtain the clean PPG ***y****_ZLE_* as follows:

(11) $$y_{ZLE}=u-w$$

where ***u*** = [*u*[0], *u*[1], *u*[2], … *u*[*N*−1]]^T^ and ***w*** = [*w*[0], *w*[1], *w*[2], … *w*[*N*−1]]^T^. *N* is the length of the input sequence. As a result, we can construct the ZLE, which combines the time-variant forward-backward harmonic notch filter with a frequency estimator, as seen in Figure 5. In this study, we employed the ALNF as the frequency estimator.

### 2.3. Real-Time Implementation of Zero-Phase Line Enhancer 

**Figure 6:**
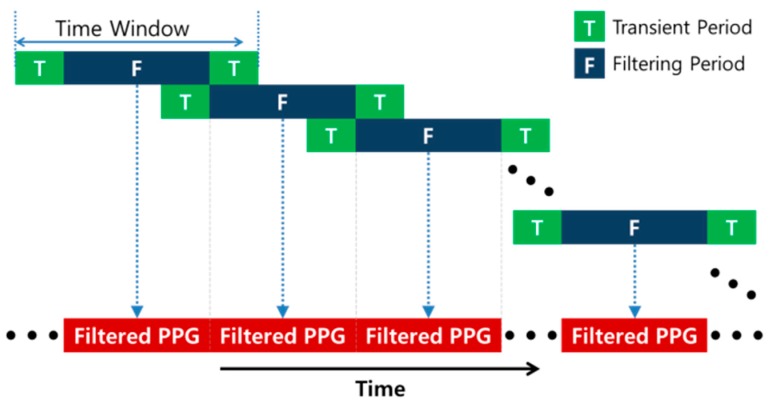
Real-time implementation scheme for ZLE algorithm.

In u-healthcare applications, real-time capability is one of the most important requirements. However, because the ZLE is a batch processor and requires the entire input data, it is necessary to design a scheme for real-time implementation. For that reason, we employed a moving window technique as shown in Figure 6. Forward-backward filtering demands transient periods at the beginning and the end of the signal. Thus, we discarded these transient periods and kept the filtering periods only. Specifically, because transient phenomena appear in both ends of the signal, the overlap duration between two consecutive time windows is two times the duration of the transient period. We chose 100 samples and 300 samples as the duration of the transient period and the filtering period, respectively. Finally, we implemented the real time ZLE system, and a demo video file for the real time implementation is attached as a supplementary material (Video S1).

### 2.4. Performance Comparison under Colored Noise Interference

**Figure 7:**
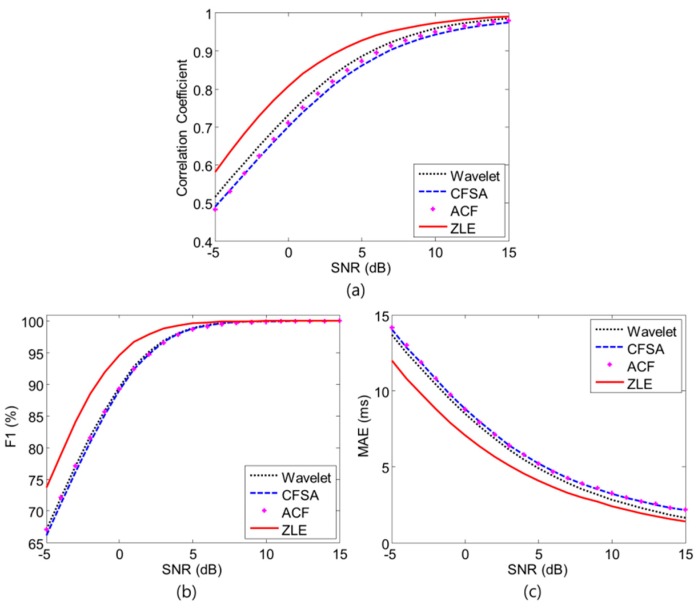
Simulation results. Dotted line, dashed line, asterisks, and solid line represent the results of wavelet de-noising method, CFSA, ACF and ZLE, respectively. (**a**) Correlation coefficient; (**b**) F1; (**c**) MAE.

In real-world situations, because the MA is mostly distributed within the feasible HR range, we generated the simulation signal by adding colored noise with a 0.5~2 Hz spectrum to the clean PPG [30]. We compared the ZLE with the ACF and two conventional single-input MA reduction methods: wavelet de-noising [16] and CFSA [17]. For the wavelet de-noising method, we adopted a biorthogonal wavelet and decomposed input signal into 7 level wavelet coefficients. The hard thresholding rule was employed and the wavelet coefficients at the last level were assigned to zero because the MA corresponds to low frequency noise. The correlation coefficient between clean PPG and filtered PPG reconstructed by each MA reduction method were used to measure the MA reduction performance. For additional performance evaluation, we measured peak accuracy. We used Matlab function ‘findpeaks’ to get peak locations of filtered PPG and clean PPG. The clean PPG’s peaks were utilized as reference peaks. Among the peaks of filtered PPG, we only accepted some peaks which fell within ±75 ms of the reference peak locations [31], and the others were rejected as missed detection. By this peak detection process, we computed some performance measures such as sensitivity (Se), positive predictive value (PPV), F1 score, and mean absolute error (MAE) [32] as follows:

(12) $$\mathrm{Se}=\frac{N\left(TP\right)}{N\left(TP\right)+N\left(FN\right)}\times 100$$

(13) $$\mathrm{PPV}=\frac{N\left(TP\right)}{N\left(TP\right)+N\left(FP\right)}\times 100$$

(14) $$F1=\frac{2\cdot N\left(TP\right)}{2\cdot N\left(TP\right)+N\left(FN\right)+N\left(FP\right)}\times 100$$

(15) $$\mathrm{MAE}=\frac{1}{N\left(TP\right)}\underset{i\in TP}{\sum }\left|{\hat{p}}_{i}-p_{i}\right|$$

where *TP*, *FP*, and *FN* represent the number of true positive, false positive, and false negative, respectively. *N*() means the number of indices. $p_{i}$ and ${\hat{p}}_{i}$ are the location of reference peak and that of the peak detected from the filtered PPG, respectively. F1 score is a measure of peak detection rate and MAE indicates peak accuracy. To clarify the comparison, Monte Carlo simulations were performed over 1000 independent simulations under a fixed input SNR. We investigated the performance trend according to different SNRs from −5 to 15 dB with an interval of 1 dB, as shown in Figure 7. The ZLE was superior to other single-input MA reduction methods. For the validation process, we did ANOVA tests for correlation coefficient, MAE, and F1 score. All *p*-values were less than 0.001 with respect to all SNR (−5 dB~15 dB). For post hoc analysis, we did one tailed *t*-test, and there were extremely significant differences between ZLE and other methods (*p* < 0.001) with respect to MAE as well as correlation coefficient for all input SNR from −5 to 15 dB. With regard to F1 score, there were extremely significant differences (*p* < 0.001) in low SNRs (<12 dB) but significant differences were not found for some case in high SNRs because most methods found peaks well without interference (SNR > 12 dB).

### 2.5. Data Acquisition

We collected PPG data with two reflection-type finger PPG sensors (BIOPAC^®^ PPG100C) from one female and seven male subjects (27.1 ± 3.4). Two PPG sensors were mounted on the right and left index fingers, and all subjects were instructed to move their right hand randomly to measure the contaminated PPG and to hold their left hand motionless for the reference PPG. Eight subjects participated in the experiment with 3 different experimental conditions: finger bending, elbow bending, and arm swing. In total, 24 datasets were collected at a 100 Hz sampling frequency over 3 min, and preprocessing was performed with a bandpass filter (0.5–10 Hz) using a third-order Butterworth IIR bandpass filter. Because the frequency estimator (ALNF) requires time to converge, we excluded the initial 1000 samples (10 s) in each data set for our analysis. The institutional review board of the Gwangju Institute of Science and Technology approved all procedures in this study.

## 3. Results

**Figure 8:**
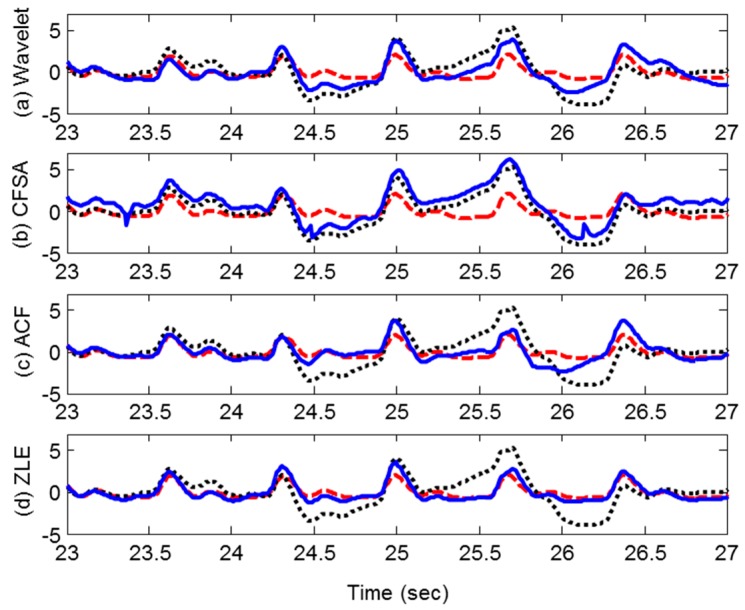
Reference, raw, and reconstructed PPG. Dashed line, dotted line, and solid line represent reference, raw, and reconstructed PPG, respectively. (**a**) Wavelet de-noising method; (**b**) CFSA; (**c**) ACF; (**d**) ZLE.

**Figure 9:**
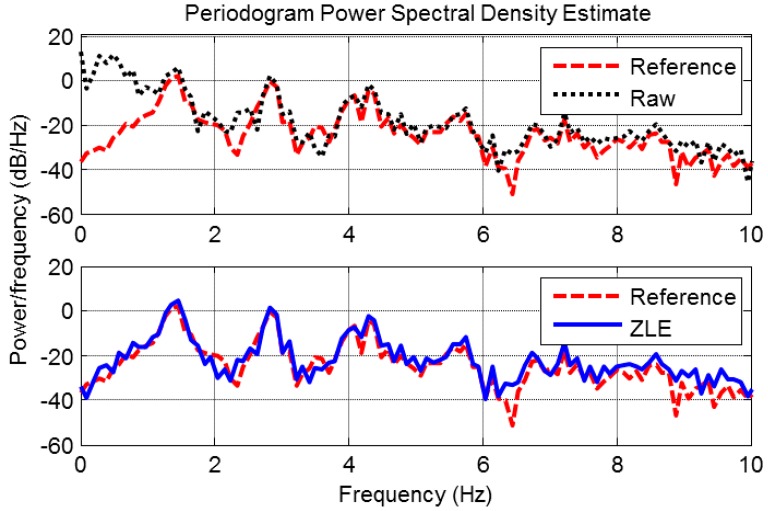
Periodogram power spectral density for PPG. Dashed line, dotted line, and solid line represent spectrums of reference, raw, and reconstructed PPG, respectively.

To verify the performance of the ZLE, we compared the ZLE with the ACF and conventional two single-input MA reduction methods: wavelet de-noising method and the CFSA. By means of these MA reduction methods, we filtered out the MA from the contaminated PPG collected from the experiment. Figure 8 depicts the filtering results. The dotted line, dashed line, and solid line correspond to the contaminated PPG, reference PPG, and the filtered PPG, respectively. Each row represents the result of a different MA reduction method. Figure 8a–d show the results of the wavelet de-noising method, the CFSA, the ACF, and the ZLE, respectively. Figure 8 indicates that the result of the ZLE traces the reference PPG much more accurately compared to the other approaches. Furthermore, the periodogram power spectral density is sufficiently recovered by the ZLE as shown in Figure 9. It is confirmed that not only the MA whose frequency range overlaps with that of HR (0.5~5 Hz) is removed well but also the fundamental frequency and its harmonic components of the PPG are well reconstructed.

**Figure 10:**
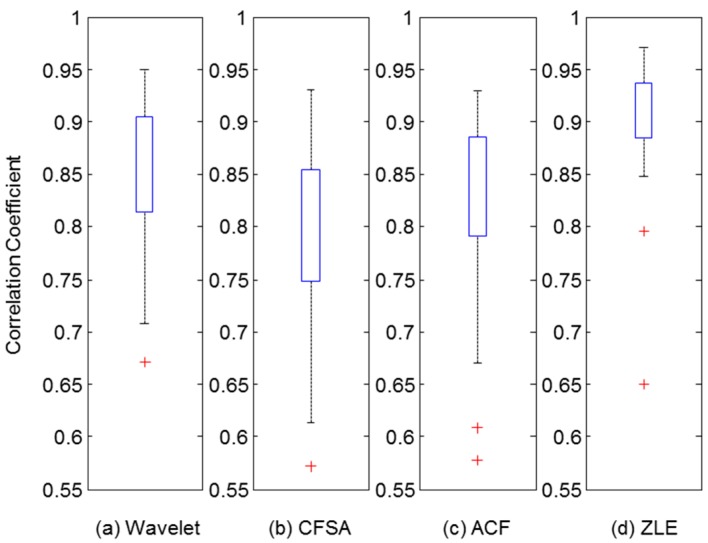
Distribution of correlation coefficient between reference and filtered PPG reconstructed by each MA reduction method. Upper and lower boxes represent the distribution of correlation coefficients from 25th to 75th percentiles. Top and bottom line indicate the 90th and 10th percentiles. (**a**) Wavelet de-noising method; (**b**) CFSA; (**c**) ACF; (**d**) ZLE.

As a performance measure, we calculated a correlation coefficient between the reference PPG and the filtered PPG in which the MA component was eliminated by each MA reduction method. Because 24 datasets were collected, 24 correlation coefficients were calculated for each MA reduction method. Figure 10 illustrates boxplots of the distribution of correlation coefficients. Upper and lower boxes represent the 75th and 25th percentiles, and the top and bottom lines indicate the 90th and 10th percentiles, respectively. Asterisks indicate outliers of the distribution of correlation coefficients. To clarify the results, we performed non-parametric Wilcoxon’s two-sampled signed rank tests between the correlation coefficients of the ZLE and those of three other methods. The Bonferroni correction was used for three multiple comparison (Wavelet vs. ZLE, CFSA vs. ZLE, and ACF vs. ZLE), and there were extremely significant differences (*p* < 0.001) for all three comparisons after Bonferroni correction. As a result, the proposed ZLE showed significantly better performance than the existing single-input MA reduction methods. Table 1 represents the descriptive statistics of correlation coefficients according to the four MA reduction methods with the three experimental conditions.

**Figure 11:**
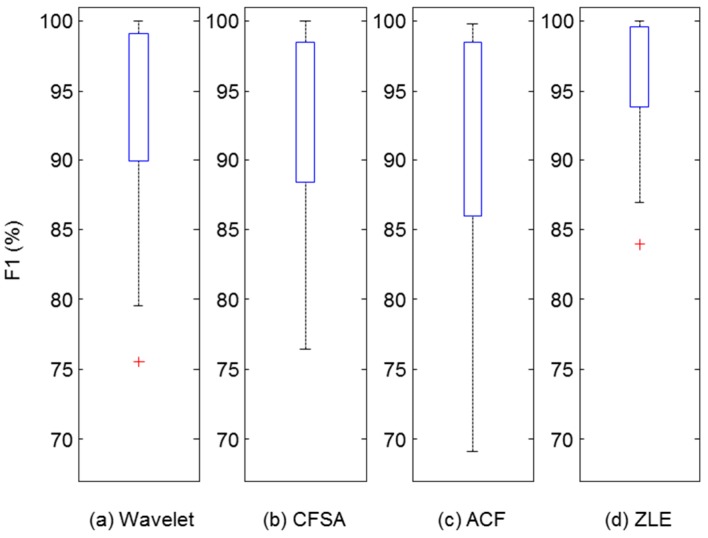
Distribution of F1 scores for filtered PPG reconstructed by each MA reduction method. Upper and lower boxes represent the distribution of correlation coefficients from 25th to 75th percentiles. Top and bottom line indicate the 90th and 10th percentiles. (**a**) Wavelet de-noising method; (**b**) CFSA; (**c**) ACF; (**d**) ZLE.

**Figure 12:**
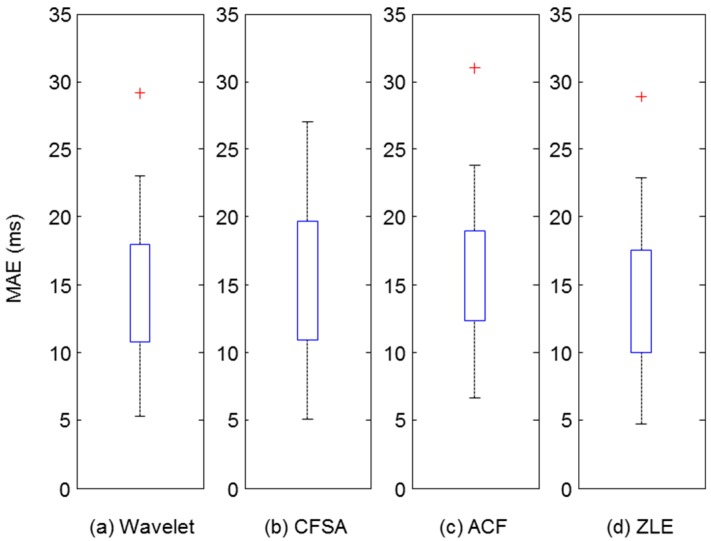
Distribution of MAE for filtered PPG reconstructed by each MA reduction method. Upper and lower boxes represent the distribution of correlation coefficients from 25th to 75th percentiles. Top and bottom line indicate the 90th and 10th percentiles. (**a**) Wavelet de-noising method; (**b**) CFSA; (**c**) ACF; (**d**) ZLE.

Many applications have used the peaks of the PPG for acquiring HR, HRV, or pulse transit time. Hence, we evaluated the accuracy of the peak location of the filtered PPG. Matlab function ‘findpeaks’ was used to find the peaks of filtered PPG and reference PPG. Among the filtered PPG’s peaks, we accepted some peaks which fell within ±75 ms of reference PPG’s peaks, and the others were rejected as missed peak detection [31]. From the results of peak detection, we computed MAE, Se, PPV, and F1 score, which were utilized as performance measures. Since the number of data sets was 24, 24 values were acquired for each performance measure (MAE, Se, PPV, and F1). Table 2 shows the descriptive statistics of these performance measures. To validate the performance, we did non-parametric Wilcoxon’s two-sampled signed rank tests between ZLE and three other methods for MAE and F1 score. We used the Bonferroni correction for multiple comparisons. With respect to F1, there was extremely significant difference (*p* < 0.001), and MAE results exhibit significant difference (*p* = 0.0164) after Bonferroni correction. Figure 11 and Figure 12 depict the distributions of F1 score and MAE, respectively. The meanings of all boxes and lines of Figure 11 and Figure 12 are the same as those of Figure 10. As a result, ZLE was found to be superior to existing single-input MA reduction methods (wavelet, CFSA, and ACF) in terms of peak detection accuracy.

## 4. Discussion

Even though many researchers have developed several techniques for MA reduction for PPG, it remains a challenge. Specifically, single-input MA reduction methods are attractive approaches for the u-healthcare system because of their low hardware costs and simplicity [15]. In this context, we proposed the ZLE, which is designed to overcome the phase distortion problem of the ACF using forward-backward filtering [29] without any additional input, and we showed that the ZLE intrinsically features the zero-phase property. Furthermore, because the ZLE is a non-causal filter, we devised a scheme for real-time implementation using a time windowing approach. For the comparison of the MA reduction methods, we performed a simulation using colored noise [30] in which the ZLE performed better than the ACF [22], CFSA [17], and wavelet [16] approaches. In addition, to verify the performance of the ZLE, we collected real PPG data contaminated by the MA with three different motions: finger bending, elbow bending, and arm swing. We compared the performance of the ZLE with those of the ACF, CFSA, and wavelet de-noising method by means of the correlation coefficient and the peak detection accuracy (F1 score, MAE), which are used as performance measures for reconstruction of the waveform. These performance measures are important because the PPG waveform is used for estimating arterial blood pressure [8,9] and SpO2, and the position of the PPG peak is used as the basic information for calculating the HR, HRV [7], and PTT. After the ZLE, the wavelet de-noising method achieved the best performance (see Figure 10, Figure 11 and Figure 12). Although it seems that ACF is better than the wavelet de-noising method in Figure 8, the actual numerical result is opposite to the result shown in Figure 8 because of the phase distortion of ACF (see Figure 10, Figure 11 and Figure 12). The CFSA can remove the MA through the integrals of Fourier series, but the interval of the integrals is too short to eliminate low frequency MA. Overall, the ZLE is significantly superior to the ACF, the CFSA, and the wavelet de-noising method with respect to its correlation coefficient as well as peak detection accuracy (F1 score, MAE) as shown in Figure 10, Figure 11 and Figure 12. In conclusion, with the introduction of time-variant forward-backward filtering, we dramatically enhanced the performance of the ACF, and this may be the first report on the linear time-variant (LTV) forward-backward IIR filter in the range of our knowledge. Although the ZLE does not offer the on-line processing property of ACF, the ZLE can process the data in real-time substantially because of its low computational complexity *O*(*N*) where *N* means the length of the input signal. The computational complexity of wavelet de-noising is *O*(*N*log*N*) [33] and that of others (CFSA, ACF, and ZLE) is *O*(*N*).

The performance of the ZLE is determined by the accuracy of the estimated instantaneous HR *θ*(*n*), and the appropriate value of *δ* in Equation (2) depends on the accuracy of *θ*(*n*). For example, if *θ*(*n*) is inaccurate, then the signal should be filtered with wide frequency range, so that the value of *δ* should be large (see Figure 4). Therefore, estimating instantaneous HR with high fidelity and finding an appropriate value of *δ* are most important for ZLE implementation.

## 5. Conclusions

We proposed the ZLE to reduce the MA from the PPG. Because ZLE not only shows remarkable MA reduction performance but also requires only a single-input without additional reference signals, we expect the ZLE to be broadly applicable to u-healthcare devices. In future work we will implement the ZLE on a hardware platform using field-programmable gate array (FPGA) and apply the ZLE to other signals such as functional near-infrared spectroscopy (fNIRs), ballistocardiogram (BCG), and electrocardiogram (ECG), and design an improved frequency estimator to estimate instantaneous HR precisely.

## Figures and Tables

**Table 1:** Correlation coefficients according to motions and methods. Data are expressed as mean ± standard deviation.

	Wavelet	CFSA	ACF	ZLE
Finger Bending	0.8237 ± 0.0716	0.7405 ± 0.0945	0.7824 ± 0.1082	0.8728 ± 0.0976
Elbow Bending	0.8777 ± 0.0548	0.8357 ± 0.0569	0.8619 ± 0.0523	0.9262 ± 0.0287
Arm Swing	0.8352 ± 0.0822	0.7956 ± 0.0929	0.8198 ± 0.1000	0.9043 ± 0.0526
Total	0.8455 ± 0.0713	0.7906 ± 0.0890	0.8214 ± 0.0924	0.9011 ± 0.0670

**Table 2:** Peak detection results for each method. Data are expressed as mean ± standard deviation.

	Wavelet	CFSA	ACF	ZLE
MAE (ms)	1.5078 ± 0.5645	1.5400 ± 0.5570	1.5582 ± 0.5499	1.4371 ± 0.5625
Se (%)	93.1247 ± 7.2786	91.5446 ± 8.1233	89.8683 ± 8.7489	96.2713 ± 4.4469
PPV (%)	93.8799 ± 6.5653	93.6469 ± 6.3911	91.8527 ± 7.7115	96.5478 ± 4.1635
F1 (%)	93.4971 ± 6.9150	92.5655 ± 7.2395	90.8341 ± 8.1817	96.4068 ± 4.2807

## Supplementary Materials

The following are available online at http://www.mdpi.com/1424-8220/17/4/860/s1. Video S1: Real-time implementation for ZLE.

## Abbreviations

The following abbreviations are used in this manuscript:

MA	Motion artifact
PPG	Photoplethysmogram
ECG	Electrocardiogram
HR	Heart rate
RR	Respiratory rate
HRV	Heart rate variability
ANC	Adaptive noise canceller
ICA	Independent Component Analysis
CFSA	Cycle by cycle Fourier series analysis
SSA	Singular spectrum analysis
ACF	Adaptive comb filter
IIR	Infinite impulse response
FIR	Finite impulse response
ZLE	Zero-phase line enhancer
ALNF	Adaptive lattice notch filter
MAE	Mean absolute error
Se	Sensitivity
PPV	Positive predictive value
SNR	Signal-to-noise ratio
LTV	linear time-variant
